# Obesity is positively related and tobacco smoking and alcohol consumption are negatively related to an increased risk of thyroid cancer

**DOI:** 10.1038/s41598-020-76357-y

**Published:** 2020-11-06

**Authors:** Soo-Youn An, So Young Kim, Dong Jun Oh, Chanyang Min, Songyoung Sim, Hyo Geun Choi

**Affiliations:** 1 Department of Otorhinolaryngology-Head & Neck Surgery, Thyroid/Head & Neck Cancer Center of the Dongnam Institute of Radiological & Medical Sciences (DIRAMS), Busan, Korea; 2 Department of Otorhinolaryngology-Head & Neck Surgery, CHA Bundang Medical Center, CHA University, Seongnam, Korea; 3 Department of Internal Medicine, Soonchunhyang University College of Medicine, Seoul Hospital, Seoul, Korea; 4 Hallym Data Science Laboratory, Hallym University College of Medicine, Anyang, Korea; 5 Graduate School of Public Health, Seoul National University, Seoul, Korea; 6 Department of Statistics and Institute of Statistics, Hallym University College of Medicine, Chuncheon, Korea; 7 Department of Otorhinolaryngology-Head & Neck Surgery, Hallym University College of Medicine, Anyang, Korea

**Keywords:** Cancer, Oncology

## Abstract

The purpose of this study was to evaluate the relationships of smoking, alcohol consumption, and obesity with thyroid cancer in Korean residents. The Korean National Health Insurance Service-Health Screening Cohort includes individuals ≥ 40 years who were assessed from 2002 to 2013. In total, 4977 thyroid cancer participants were matched with respect to age, sex, income, and region of residence with 19,908 controls at a ratio of 1:4. Crude and adjusted (for the Charlson comorbidity index, smoking status, frequency of alcohol consumption, and obesity) odds ratios (ORs) were analyzed using conditional logistic regression analyses. Additionally, 95% confidence intervals (CIs) were calculated. The adjusted OR of smoking for thyroid cancer was 0.62 (95% CI 0.54–0.72, P < 0.001), and that of alcohol consumption was 0.83 (95% CI 0.75–0.92, P < 0.001). The adjusted ORs of the BMI categories were 1.13 (95% CI 1.05–1.22, P = 0.002) for obese I, and 1.24 (95% CI 1.04–1.47, P = 0.014) for obese II. The ORs of smoking and alcohol consumption were lower, and those of overweight and obesity were higher in thyroid cancer patients than in individuals in the control group.

## Introduction

The incidence of thyroid cancer is increasing in many countries^1,2^. Specifically, in South Korea, the incidence of thyroid cancer increased 15-fold between 1993 and 2011^3^, and the age-standardized incidence of thyroid cancer per 100,000 people was 23.0 in men and 102.4 in women in 2012^4^. A higher rate of overdiagnosis as a result of health screening with ultrasonography is apparently responsible for this^3,5^. However, it is not the only explanation of the increasing incidence of thyroid cancer^6^. Some etiologic factors, including obesity, might affect the increasing incidence of thyroid cancer^7^. Other conditions such as radiation exposure and iodine intake are also considered risk factors for thyroid cancer^1^. However, these contributions remain speculative.

Previously, several studies reported associations of obesity, smoking, and drinking alcohol with thyroid cancer^7–9^. However, the numbers of included participants were relatively few (< 1000 cases of thyroid cancer) in most studies. Few studies have analyzed obesity, smoking, and alcohol consumption in a single study after adjusting for the effects of these variables even though they are closely related.

The purpose of this study was to identify the factors that predispose individuals to thyroid cancer using a national health checkup cohort of South Korean residents. In this study, we analyzed the odds ratios (ORs) of obesity, smoking, and alcohol consumption in terms of thyroid cancer compared to the 1:4 matched control group. In this study, we focused on these factors because they could be modifiable and controlling them could affect the incidence of thyroid cancer.

## Results

The rates of smoking and alcohol consumption were lower in the thyroid cancer group (smoking = 5.5% [273/4977]; alcohol consumption = 11.8% [598/4977]) than in the control group (smoking = 8.1% [1618/19,908]; alcohol consumption = 14.0% [2791/19,908]; each P =  < 0.001, Table 1). The thyroid cancer group included more obese participants than the control group (P < 0.001). The general characteristics (age, sex, income, and region of residence) of the participants were the same due to the matching procedure (P = 1.000). The CCI score was different between the thyroid cancer and control groups (P < 0.001).

**Table 1:** General participant characteristics.

Characteristics	Total participants		
	Thyroid cancer (n, %)	Control (n, %)	P-value
**Age (years)**			1.000
40–44	104 (2.1)	416 (2.1)	
45–49	797 (16.0)	3188 (16.0)	
50–54	1427 (28.7)	5708 (28.7)	
55–59	1084 (21.8)	4336 (21.8)	
60–64	727 (14.6)	2908 (14.6)	
65–69	476 (9.6)	1904 (9.6)	
70–74	268 (5.4)	1072 (5.4)	
75–79	74 (1.5)	296 (1.5)	
80–84	19 (0.4)	76 (0.4)	
85 +	1 (0.0)	4 (0.0)	
**Sex**			1.000
Male	1030 (20.7)	4120 (20.7)	
Female	3947 (79.3)	15,788 (79.3)	
**Income**			1.000
1 (lowest)	609 (12.2)	2436 (12.2)	
2	562 (11.3)	2248 (11.3)	
3	762 (15.3)	3048 (15.3)	
4	984 (19.8)	3936 (19.8)	
5 (highest)	2060 (41.4)	8240 (41.4)	
**Region of residence**			1.000
Urban	2418 (48.6)	9672 (48.6)	
Rural	2559 (51.4)	10,236 (51.4)	
**Charlson comorbidity index**			< 0.001*
0	4799 (96.4)	19,617 (98.5)	
1	0 (0.0)	48 (0.2)	
2	47 (0.9)	59 (0.3)	
3	14 (0.3)	51 (0.3)	
≥ 4	117 (2.4)	133 (0.7)	
**Obesity (BMI, kg/m**^**2**^**)**			< 0.001*
< 18.5 (underweight)	61 (1.2)	350 (1.8)	
≥ 18.5 to < 23 (normal)	1662 (33.4)	7097 (35.6)	
≥ 23 to < 25 (overweight)	1420 (28.5)	5608 (28.2)	
≥ 25 to < 30 (obese I)	1643 (33.0)	6196 (31.1)	
≥ 30 (obese II)	191 (3.8)	657 (3.3)	
**Smoking status**			< 0.001*
Nonsmoker or past smoker	4704 (94.5)	18,290 (91.9)	
Current smoker	273 (5.5)	1618 (8.1)	
**Alcohol consumption**			< 0.001*
< 1 time a week	4388 (88.2)	17,117 (86.0)	
≥ 1 time a week	589 (11.8)	2791 (14.0)	

*BMI* body mass index, kg/m^2^.

*Chi-square test. Significance at P < 0.05.

The adjusted OR (aOR) of smoking for thyroid cancer was 0.62 (95% CI 0.54–0.72, P < 0.001), and that of alcohol consumption was 0.83 (95% CI 0.75–0.92, P < 0.001, Table 2). The aORs of the BMI categories were 0.75 (95% CI 0.57–0.99, P = 0.042) for underweight, 1.08 (95% CI 1.00–1.17, P = 0.050) for overweight, 1.13 (95% CI 1.05–1.22, P = 0.002) for obese I, and 1.24 (95% CI 1.04–1.47, P = 0.014) for obese II compared to a normal BMI in the thyroid cancer group.

**Table 2:** Crude and adjusted odd ratios (95% confidence intervals) of smoking, alcohol consumption, and obesity for thyroid cancer.

Characteristics	N of thyroid cancer	N of control	ORs of thyroid cancer			
	(Exposure/total, %)	(Exposure/total, %)	Crude^†^	P-value	Adjusted^†^^‡^	P-value
Smoking status	273/4977 (5.5%)	1618/19,908 (8.1%)	0.60 (0.52–0.69)	< 0.001*	0.62 (0.54–0.72)	< 0.001*
Alcohol consumption	589/4977 (11.8%)	2791/19,908 (14.0%)	0.79 (0.71–0.88)	< 0.001*	0.83 (0.75–0.92)	< 0.001*
Obesity (BMI, kg/m^2^)				< 0.001*		< 0.001*
< 18.5 (underweight)	61/4977 (1.2%)	350/19,908 (1.8%)	0.74 (0.56–0.98)	0.037*	0.75 (0.57–0.99)	0.042*
≥ 18.5 to < 23 (normal)	1662/4977 (33.4%)	7097/19,908 (35.7%)	1.00		1.00	
≥ 23 to < 25 (overweight)	1420/4977 (28.5%)	5608/19,908 (28.2%)	1.09 (1.00–1.17)	0.045*	1.08 (1.00–1.17)	0.050*
≥ 25 to < 30 (obese I)	1643/4977 (33.0%)	6196/19,908 (31.1%)	1.14 (1.05–1.23)	0.001*	1.13 (1.05–1.22)	0.002*
≥ 30 (obese II)	191/4977 (3.8%)	657/19,908 (3.3%)	1.25 (1.05–1.48)	0.011*	1.24 (1.04–1.47)	0.014*

*Conditional logistic regression analysis, Significance at P < 0.05.

^†^Stratified model for age, sex, income, and region of residence.

^‡^Adjusted model for Charlson comorbidity index, obesity, smoking state (current smoker compared to nonsmoker or past smoker) and frequency of alcohol consumption (≥ 1 time a week compared to < 1 time a week).

In subgroup analyses performed according to age and sex, the aOR of obesity was statistically significant in individuals ≥ 55 years and in men. The aORs of smoking reached statistical significance in all groups, while alcohol consumption was significant in individuals < 55 years and ≥ 55 years and in women (Table 3).

**Table 3:** Crude and adjusted odd ratios (95% confidence interval) of smoking, alcohol consumption, and obesity for thyroid cancer in each stratified group according age and sex.

Characteristics	N of thyroid cancer	N of tontrol	ORs of thyroid cancer			
	(Exposure/total, %)	(Exposure/total, %)	Crude^†^	P-value	Adjusted^†^^‡^	P-value
** < 55 years (n = 11,640)**						
Smoking status	164/2328 (7.0%)	871/9312 (9.4%)	0.68 (0.59–0.82)	< 0.001*	0.71 (0.58–0.86)	< 0.001*
Alcohol consumption	321/2328 (13.8%)	1547/9312 (16.6%)	0.77 (0.67–0.89)	< 0.001*	0.80 (0.69–0.92)	0.002*
Obesity (BMI, kg/m^2^)				0.145		0.133
< 18.5 (underweight)	32/2328 (1.4%)	174/9312 (1.9%)	0.77 (0.52–1.13)	0.182	0.79 (0.54–1.16)	0.235
≥ 18.5 to < 23 (normal)	895/2328 (38.5%)	3749/9312 (40.3%)	1.00		1.00	
≥ 23 to < 25 (overweight)	660/2328 (28.4%)	2574/9312 (27.6%)	1.08 (0.96–1.21)	0.199	1.08 (0.96–1.21)	0.186
≥ 25 to < 30 (obese I)	668/2328 (28.7%)	2565/9312 (27.6%)	1.10 (0.98–1.23)	0.116	1.09 (0.97–1.22)	0.136
≥ 30 (obese II)	73/2328 (3.1%)	250/9312 (2.7%)	1.23 (0.94–1.61)	0.138	1.21 (0.92–1.59)	0.175
** ≥ 55 year (n = 13,245)**						
Smoking	109/2649 (4.1%)	747/10,596 (7.1%)	0.52 (0.42–0.64)	< 0.001*	0.53 (0.42–0.66)	< 0.001*
Alcohol consumption	268/2649 (10.1%)	1244/10,596 (11.7%)	0.82 (0.70–0.95)	0.009*	0.86 (0.74–1.00)	0.056
Obesity (BMI, kg/m^2^)				0.005*		0.006*
< 18.5 (underweight)	29/2649 (1.1%)	176/10,596 (1.7%)	0.72 (0.48–1.07)	0.107	0.72 (0.48–1.08)	0.109
≥ 18.5 to < 23 (normal)	767/2649 (29.0%)	3348/10,596 (31.6%)	1.00		1.00	
≥ 23 to < 25 (overweight)	760/2649 (28.7%)	3034/10,596 (28.6%)	1.10 (0.98–1.22)	0.114	1.09 (0.97–1.22)	0.141
≥ 25 to < 30 (obese I)	975/2649 (36.8%)	3631/10,596 (34.3%)	1.17 (1.06–1.31)	0.003*	1.16 (1.05–1.29)	0.005*
≥ 30 (obese II)	118/2649 (4.5%)	407/10,596 (3.8%)	1.27 (1.02–1.58)	0.035*	1.25 (1.01–1.56)	0.045*
**Men (n = 5150)**						
Smoking	232/1030 (22.5%)	1291/4120 (31.3%)	0.63 (0.54–0.74)	< 0.001*	0.65 (0.55–0.76)	< 0.001*
Alcohol consumption	385/1030 (37.4%)	1744/4120 (42.3%)	0.82 (0.71–0.94)	0.004*	0.85 (0.73–0.98)	0.024*
Obesity (BMI, kg/m^2^)				< 0.001*		< 0.001*
< 18.5 (underweight)	8/1030 (0.8%)	59/4120 (1.4%)	0.69 (0.32–1.46)	0.330	0.67 (0.31–1.44)	0.302
≥ 18.5 to < 23 (normal)	232/1030 (22.5%)	1188/4120 (28.8%)	1.00		1.00	
≥ 23 to < 25 (overweight)	323/1030 (31.4%)	1264/4120 (30.7%)	1.32 (1.09–1.59)	0.004*	1.31 (1.08–1.58)	0.006*
≥ 25 to < 30 (obese I)	429/1030 (41.7%)	1,513/4,120 (36.7%)	1.46 (1.22–1.74)	< 0.001*	1.44 (1.20–1.72)	< 0.001*
≥ 30 (obese II)	38/1,030 (3.7%)	96/4,120 (2.3%)	2.05 (1.37–3.06)	< 0.001*	2.01 (1.34–3.03)	< 0.001*
**Women (n = 19,735)**						
Smoking	41/3947 (1.0%)	327/15,788 (2.1%)	0.50 (0.36–0.69)	< 0.001*	0.53 (0.38–0.74)	< 0.001*
Alcohol consumption	204/3947 (5.2%)	1047/15,788 (6.6%)	0.77 (0.66–0.89)	< 0.001*	0.80 (0.69–0.94)	0.006*
Obesity (BMI, kg/m^2^)				0.088		0.113
< 18.5 (underweight)	53/3947 (1.3%)	291/15,788 (1.8%)	0.75 (0.56–1.02)	0.062	0.76 (0.57–1.03)	0.077
≥ 18.5 to < 23 (normal)	1430/3947 (36.2%)	5909/15,788 (37.4%)	1.00		1.00	
≥ 23 to < 25 (overweight)	1097/3947 (27.8%)	4344/15,788 (27.5%)	1.05 (0.96–1.14)	0.328	1.05 (0.96–1.14)	0.329
≥ 25 to < 30 (obese I)	1214/3947 (30.8%)	4683/15,788 (29.7%)	1.07 (0.99–1.17)	0.107	1.07 (0.98–1.17)	0.116
≥ 30 (obese II)	153/3947 (3.9%)	561/15,788 (3.6%)	1.13 (0.94–1.37)	0.202	1.12 (0.93–1.36)	0.225

*Conditional logistic regression analysis, Significance at P < 0.05.

^†^Stratified model for age, sex, income, and region of residence.

^‡^Adjusted model for Charlson comorbidity index, obesity, smoking state (current smoker compared to nonsmoker or past smoker) and frequency of alcohol consumption (≥ 1 time a week compared to < 1 time a week).

We analyzed the ORs of thyroid cancer in the subgroups of nonsmokers or past smokers, current smokers, individuals who consume alcohol < 1 time a week, individuals who consume alcohol ≥ 1 time a week, patients who are underweight, patients who are normal weight, individuals who are overweight, individuals in the obese I group, and individuals in the obese II group (Table 4). Although these subgroup analyses did not follow the matched structure of this study, the results were consistent with the main results. These associations did not reach statistical significance in the underweight or obese II groups due to the relatively small number of participants.

**Table 4:** Odd ratios (95% confidence interval) of smoking, alcohol consumption, and obesity for thyroid cancer in each group.

Characteristics	N of thyroid cancer	N of control	ORs of thyroid cancer			
	(Exposure/total, %)	(Exposure/total, %)	Crude	P-value	Adjusted^†^	P-value
**Non or past smoker (n = 22,994)**						
Alcohol consumption	456/4704 (9.7%)	2003/18,290 (11.0%)	0.87 (0.78–0.97)	0.013*	0.81 (0.72–0.91)	< 0.001*
Obesity (BMI, kg/m^2^)				0.004*		0.004*
< 18.5 (underweight)	58/4704 (1.2%)	309/18,290 (1.7%)	0.78 (0.58–1.03)	0.084	0.77 (0.58–1.02)	0.072
≥ 18.5 to < 23 (normal)	1588/4704 (33.8%)	6572/18,290 (35.9%)	1.00		1.00	
≥ 23 to < 25 (overweight)	1344/4704 (28.6%)	5123/18,290 (28.0%)	1.09 (1.00–1.18)	0.047*	1.08 (1.00–1.18)	0.054
≥ 25 to < 30 (obese I)	1533/4704 (32.6%)	5669/18,290 (31.0%)	1.12 (1.03–1.21)	0.005*	1.11 (1.03–1.21)	0.008*
≥ 30 (obese II)	181/4704 (3.9%)	617/18,290 (3.4%)	1.21 (1.02–1.45)	0.029*	1.22 (1.02–1.45)	0.027*
**Current smoker (n = 1891)**						
Alcohol consumption	133/273 (48.7%)	788/1618 (48.7%)	1.00 (0.77–1.29)	0.996	0.93 (0.71–1.21)	0.565
Obesity (BMI, kg/m^2^)				0.046*		0.080
< 18.5 (underweight)	3/273 (1.1%)	41/1618 (2.5%)	0.52 (0.16–1.72)	0.283	0.57 (0.17–1.90)	0.360
≥ 18.5 to < 23 (normal)	74/273 (27.1%)	525/1618 (32.5%)	1.00		1.00	
≥ 23 to < 25 (overweight)	76/273 (27.8%)	485/1618 (30.0%)	1.11 (0.79–1.57)	0.545	1.05 (0.74–1.49)	0.779
≥ 25 to < 30 (obese I)	110/273 (40.3%)	527/1618 (32.6%)	1.48 (1.08–2.04)	0.016*	1.44 (1.05–2.00)	0.026*
≥ 30 (obese II)	10/273 (3.7%)	40/1618 (2.5%)	1.77 (0.85–3.70)	0.126	1.60 (0.75–3.42)	0.222
**Consuming alcohol < 1 time a week (n = 21,505)**						
Smoking	140/4388 (3.2%)	830/17,117 (4.9%)	0.65 (0.54–0.78)	< 0.001*	0.58 (0.47–0.70)	< 0.001*
Obesity (BMI, kg/m^2^)				0.003*		0.004*
< 18.5 (underweight)	52/4388 (1.2%)	303/17,117 (1.8%)	0.71 (0.52–0.95)	0.023*	0.71 (0.52–0.95)	0.024*
≥ 18.5 to < 23 (normal)	1522/4388 (34.7%)	6256/17,117 (36.6%)	1.00		1.00	
≥ 23 to < 25 (overweight)	1229/4388 (28.0%)	4782/17,117 (27.9%)	1.06 (0.97–1.15)	0.201	1.06 (0.97–1.15)	0.211
≥ 25 to < 30 (obese I)	1427/4388 (32.5%)	5212/17,117 (30.5%)	1.13 (1.04–1.22)	0.004*	1.12 (1.03–1.22)	0.006*
≥ 30 (obese II)	158/4388 (3.6%)	564/17,117 (3.3%)	1.15 (0.96–1.39)	0.135	1.15 (0.96–1.39)	0.136
**Consuming alcohol ≥ 1 time a week (n = 3380)**						
Smoking	133/589 (22.6%)	788/2791 (28.2%)	0.74 (0.60–0.92)	0.005*	0.68 (0.54–0.85)	< 0.001*
Obesity (BMI, kg/m^2^)				0.005*		0.030*
< 18.5 (underweight)	9/589 (1.5%)	47/2791 (1.7%)	1.15 (0.55–2.40)	0.709	1.17 (0.55–2.48)	0.682
≥ 18.5 to < 23 (normal)	140/589 (23.8%)	841/2791 (30.1%)	1.00		1.00	
≥ 23 to < 25 (overweight)	191/589 (32.4%)	826/2791 (29.6%)	1.39 (1.10–1.76)	0.007*	1.32 (1.03–1.68)	0.026*
≥ 25 to < 30 (obese I)	216/589 (36.7%)	984/2791 (35.3%)	1.32 (1.05–1.66)	0.019*	1.24 (0.98–1.56)	0.078
≥ 30 (obese II)	33/589 (5.6%)	93/2791 (3.3%)	2.13 (1.38–3.30)	< 0.001*	1.95 (1.25–3.05)	0.003*
**Underweight (BMI < 18.5, n = 411)**						
Smoking	3/61 (4.9%)	41/350 (11.7%)	0.39 (0.12–1.30)	0.126	0.34 (0.09–1.28)	0.111
Alcohol consumption	9/61 (14.8%)	47/350 (13.4%)	1.12 (0.52–2.41)	0.781	1.56 (0.64–3.77)	0.327
**Normal weight (BMI ≥ 18.5 to < 23, n = 8759)**						
Smoking	74/1662 (4.5%)	525/7097 (7.4%)	0.58 (0.46–0.75)	< 0.001*	0.65 (0.49–0.85)	0.002*
Alcohol consumption	140/1662 (8.4%)	841/7097 (11.9%)	0.69 (0.57–0.83)	< 0.001*	0.77 (0.63–0.94)	0.012*
**Overweight (BMI ≥ 23 to < 25, n = 7028)**						
Smoking	76/1420 (5.4%)	485/5608 (8.7%)	0.60 (0.47–0.77)	< 0.001*	0.52 (0.40–0.69)	< 0.001*
Alcohol consumption	191/1420 (13.5%)	826/5608 (14.7%)	0.90 (0.76–1.07)	0.222	0.93 (0.77–1.12)	0.416
**Obese I (BMI ≥ 25 to < 30, n = 7839)**						
Smoking	110/1643 (6.7%)	527/6196 (8.5%)	0.77 (0.62–0.96)	0.017*	0.71 (0.57–0.90)	0.004*
Alcohol consumption	216/1643 (13.2%)	984/6196 (15.9%)	0.80 (0.68–0.94)	0.006*	0.74 (0.62–0.89)	< 0.001*
**Obese II (BMI ≥ 30, n = 848)**						
Smoking	10/191 (5.2%)	40/657 (6.1%)	0.85 (0.42–1.74)	0.660	0.57 (0.25–1.27)	0.168
Alcohol consumption	33/191 (17.3%)	93/657 (14.2%)	1.27 (0.82–1.96)	0.286	1.09 (0.66–1.80)	0.749

*Unconditional logistic regression analysis, Significance at P < 0.05.

^†^Adjusted model for age, sex, income, region of residence, Charlson comorbidity index, obesity, smoking state (current smoker compared to nonsmoker or past smoker) and frequency of alcohol consumption (≥ 1 time a week compared to < 1 time a week).

## Discussion

In the present study, participants with thyroid cancer had higher ORs for overweight and obese and lower ORs for smoking and alcohol consumption than participants in the control group. These findings were consistent in the subgroups of age and sex.

Obesity could affect thyroid tumorigenesis through various mechanisms^7^. Obesity results in (i) hyperinsulinemia with an increase in IGF-1, (ii) an increase in leptin and a decrease in adiponectin, (iii) chronic inflammation via IL-6, TNF-α, PAI-1, and NF-κB, (iv) an increase in free fatty acids, and (v) oxidative stress including DNA damage^10^. Most previous studies have shown that obesity is associated with thyroid cancer, while a few studies exhibited inconsistent results. A previous large meta-analysis (n of thyroid cancer patients = 3587) reported that each 5 kg/m^2^ increase in BMI increased thyroid cancer risk 1.33 times (95% CI 1.04–1.70) in men and 1.14 times (95% CI 1.06–1.23) in women^11^. Another cohort study reported that the relative risk (RR) for thyroid cancer was 1.89 (95% CI 1.21–2.96) in men with a BMI ≥ 30.0 kg/m^2^, while the RR was 1.10 (95% CI 0.75–1.61) in women with a BMI ≥ 30.0 kg/m^2^^12^. Another cohort study exhibited contrary results of a hazard ratio (HR) of 2.14 (95% CI 0.06–7.67) in men with a BMI ≥ 35.0 kg/m^2^ and an HR of 1.74 (95% CI 1.03–2.94) in women with a BMI ≥ 35.0 kg/m^2^^13^. Some studies did not reveal any relationship between obesity and thyroid cancer (a cohort study in 432 participants)^14^. However, that study was based on a relatively small study population of 432 participants and analyzed the association of obesity with more aggressive features of differentiated thyroid cancer^15^. Another previous study even found a negative association in single hospital-based study^16^. However, that study also had small study population (253 patients) and investigated the relation of obesity with the risk of thyroid cancer in patients with indeterminate thyroid nodules^16^. We found that obesity was associated with thyroid cancer in the ≥ 55-year-old group and in men using the Asian BMI classification^17^. Although the P values of obesity in women and the < 55-year-old group did not reach statistical significance, their ORs showed an increasing trend as BMI increased. Differences in the effects of estrogen from adipose tissue with different aromatization might cause these differences with regard to age and sex^18^. In Korea, the rate of obesity (≥ 25 kg/m^2^) increased from 25.1% in 1998 to 42.3% in 2016 in men, while the rate was stable at approximately 26.2–26.4% during the same time period in women^19^. The abrupt increase in obesity in men might affect the difference between men and women. Unconsidered factors such as dietary and exercise habits, which are related to obesity, may be involved in this difference.

Relatively consistently, smoking has been reported as a factor that reduces the risk of thyroid cancer. A pooled analysis reported ORs of 0.6 (95% CI 0.6–0.7) in current smokers and 0.9 (95% CI 0.8–1.1) in former smokers^8^. A recent meta-analysis reported an OR of 0.79 (95% CI 0.70–0.88) in ever smokers^20^. The OR of thyroid cancer was 0.49 (95% CI 0.31–0.78) in current smokers and 0.75 (95% CI 0.48–1.17) in former smokers compared to nonsmokers in a group of Korea adults^21^. Smoking decreases thyroid-stimulating hormone (TSH) levels, which has major effects on thyroid cancer development^22^. Thiocyanate, a byproduct of smoking, has shown antithyroid activity^23^. Smoking enhances the formation of inactive catechol estrogens by altering the metabolism of estradiol^24^, and estrogen has growth-promoting effects in the thyroid^25^. The relatively lower body weights of current smokers were regarded as the connection resulting in a lower risk of thyroid cancer in smokers^26^; however, we found a consistent result after adjusting for BMI in this study. Although only a few women (1.9% [368/19,367]) were current smokers (Table S1), smoking was still significantly negatively associated with thyroid cancer in women.

In previous studies, alcohol consumption was negatively associated with thyroid cancer. A meta-analysis reported an RR of 0.74 (95% CI 0.67–0.83) for the highest use of alcohol compared to the lowest use^9^. Another meta-analysis displayed an RR of 0.80 (95% CI 0.71–0.90) for > one drink/day of alcohol compared to ≤ one drink/day^27^. The OR of consuming alcohol 1–5 times a week was 0.45 (95% CI 0.24–0.84) compared to nonconsumption^28^. However, this association was eliminated after adjusting for smoking in some studies^8^, and the relationship was different according to the kinds of alcoholic beverages^29^. Some plausible hypotheses can explain this finding. Alcohol intake may reduce TSH levels. Chronic alcohol consumption changes the hypothalamic-pituitary-thyroid (HPT) axis via the action of thyrotropin-releasing hormone (TRH) neurons of the paraventricular nucleus in animal studies^30^. The toxicity of alcohol might affect thyroid cells directly and reduce thyroid volume^31^. Moderate alcohol intake improves insulin sensitivity^32^ but is linked to thyroid cancer^10^. Consuming alcohol was related to decreased sex hormone-binding globulin^33^, which might influence thyroid cancer. In this study, consuming alcohol was negatively associated with thyroid cancer, with the exception of the ≥ 55-year-old group, but this difference was not statistically significant. We also analyzed the interaction between alcohol consumption and smoking, as they were closely related (Table S2). The interaction of smoking*consuming alcohol showed statistical significance with an OR of 0.80 (95% CI 0.75–0.84) in the crude model, while it was not significant in the adjusted model. Therefore, we could not find an interaction effect of smoking and alcohol consumption on thyroid cancer.

This study has various merits including a large sample size. First, we used rigorous statistical analysis techniques such as the conditional logistics regression for the matched design. Additionally, 17 kinds of disease categories were adjusted using CCI scores. We also analyzed the interaction effects of obesity, smoking status, and alcohol consumption in a single study. Additionally, we analyzed the interactions in subgroups with respect to age, sex, obesity, smoking, and alcohol consumption using the large number of participants and maintaining statistical power. Using previous health checkup results before the diagnosis of thyroid cancer could have minimized the recall bias, which is very common in other surveyed cross-sectional studies using a questionnaire after the diagnosis of thyroid cancer. The mean time from the heath check-up to the index date was > 1 year in this study. A prior study demonstrated that the incidence of thyroid cancer in Korea was very high due to the increased incidence of diagnosis by ultrasonography, and this was more common in the higher income group^3^. Because we matched the participants according to income group, we could lessen this effect of detection bias. As obesity, smoking, and consuming alcohol could be different according to social classes and act as confounders, the matching of the subjects according to income group is an important strength of this study. Because we used the Asian-Pacific BMI classification, we calculated the OR of the relatively less obese BMI group (overweight from 23 to 25 kg/m^2^).

The limitations of this study were as follows. We used patient claim codes in the HIRA data to identify thyroid cancer. This method could lead to the underestimation of the incidence of thyroid cancer because thyroid cancer patients who did not visit clinics might be ignored. However, as stated above, the overdiagnosis of thyroid cancer is a major problem in Korea. We did not have information on the pathology of thyroid cancer, but 95% of thyroid cancer in Korea is papillary thyroid carcinoma^34^. We included only participants ≥ 40 years due to screening ages for health checkups. Despite the matching of income level, overdiagnosis in our cohort could influence to the current results. Radiation exposure, family history of thyroid cancer, dietary habits and physical activities were not surveyed in this study. Although we used survey data before the diagnosis of thyroid cancer, the chance of recall bias still exists. Because we only used health checkup data before the index date, this might be acting as a source of bias. We could not calculate the total smoking and alcohol consumption due to the categorical nature of the questionnaire. Because smoking and obesity were negatively associated, the positive association between obesity and thyroid cancer may have affected the inverse association between smoking and thyroid cancer, despite the adjustment. The relatively small fraction of current smokers and drinkers, especially women, could have affected the association of smoking or consuming alcohol with thyroid cancer in our cohort. The positive relation between and obesity was dominant in men but not in women. This might have been affected by the different contributions of obesity according to sex (Table S5). This study did not adjust for educational level or reproductive factors of women as covariates.

In conclusion, the odds of being overweight or obese were higher in the thyroid cancer group than in the control group. On the other hand, the odds of smoking and consuming alcohol were lower in the thyroid cancer group.

## Materials and methods

### Study population and data collection

The Ethics Committee of Hallym University (2017-I102) approved the use of these data. The study was exempted from the need for written informed consent by the Institutional Review Board of Hallym University. All methods were carried out in accordance with relevant guidelines and regulations.

This national cohort study relied on data from the Korean National Health Insurance Service-Health Screening Cohort (NHIS-HEALS). A detailed description of these data was described in our previous studies^35^.

#### Participant selection

**Figure 1:**
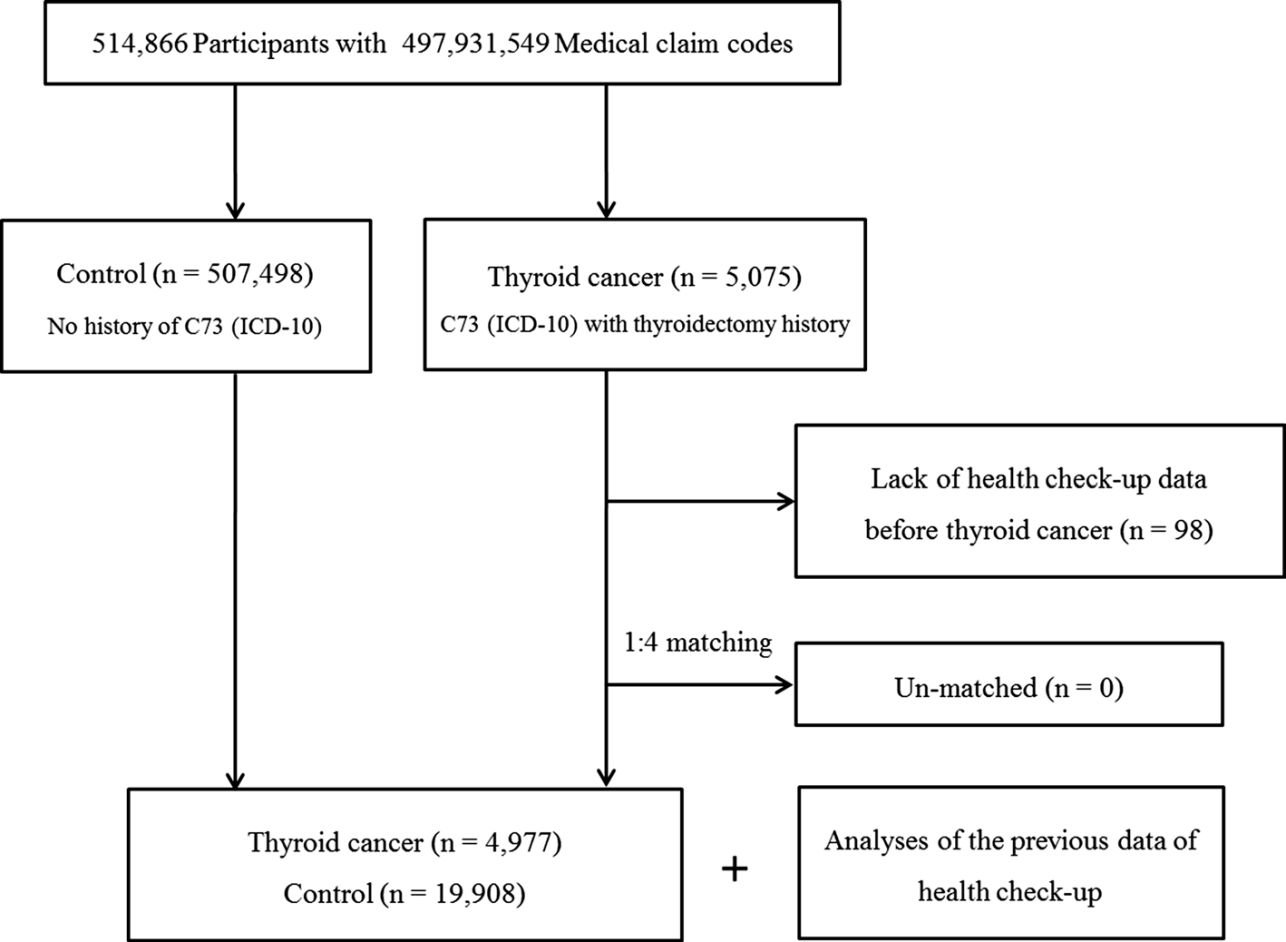
A schematic illustration of the participant selection process used in the present study. Of a total of 514,866 participants, 4977 thyroid cancer patients were matched with 19,908 control participants based on age, sex, income, and region of residence.

Out of 514,866 patients with 497,931,549 medical claim codes, we included participants who were defined as having thyroid cancer (n = 5075). The thyroid cancer participants were matched 1:4 with participants (the control group) who were never diagnosed with thyroid cancer from 2002 through 2013 among this cohort. The control group was selected from the original population (n = 507,498). Subjects were matched based on age group, sex, income group, and region of residence. Participants in the control group were sorted using a random number order and were selected from top to bottom to prevent selection bias when selecting the matched control participants. The index date was set as the date of diagnosis of thyroid cancer. It was assumed that the matched control participants were also involved in the cohort at the same time as each matched thyroid cancer participant (index date). Therefore, the control participant who died before the index date or who had no previous health check-up before the index date was replaced with another candidate. Thyroid cancer participants who had no previous health checkup before the index date were excluded (n = 98). In this study, we used the latest health checkup data before the index date. None of the thyroid cancer participants were excluded due to a lack of matched participants. The mean time from the heath checkup to the index date was 15.6 months (standard deviation [SD] = 18.3) in the thyroid cancer group and 19.5 months (SD = 20.8) in the control group. Finally, 1:4 matching resulted in the inclusion of 4977 thyroid cancer participants and 19,908 control participants. We analyzed the previous health checkup data in the thyroid cancer group and the control group after matching (Fig. 1).

### Variables

#### Independent variable

Tobacco smoking was surveyed and categorized based on the participant’s current smoking status (nonsmoker, past smoker, and current smoker), duration of smoking (nonsmoker, < 20 year, and ≥ 20 years), and current number of cigarettes smoked per day (0 cigarettes a day, < 20 cigarettes a day, and ≥ 20 cigarettes a day, Tables S3 and S4). Because every smoking category was different between the thyroid cancer group and the control group among smoking status, duration of smoking, or current number of cigarette (Table S3), we selected the smoking status of current smokers as the main variable for this study. Because the number of past smokers was small (5.9% [1462/24,885]) and we could not find a difference in thyroid cancer rates between nonsmokers and past smokers (Table S4), we merged past smokers with nonsmokers. Below, we used ‘smoking’ when referring to current smokers compared to nonsmokers or past smokers (Tables 2, 3, and 4).

Alcohol consumption was surveyed and categorized based on the frequency of alcohol consumption (< 1 time a week and ≥ 1 time a week) and the amount of alcohol consumed at a time (< soju 1 bottle, 1 soju bottle, and > 1 soju bottle, Tables S3 and S4). Because every alcohol-related category was different between the thyroid cancer group and the control group (Table S1), we selected the frequency of alcohol consumption in this study. Because the rate of thyroid cancer was not different in the nondrinkers and < 1-time-a-week group (Table S2), we merged them. Below, we used ‘alcohol consumption’ to refer to consuming alcohol ≥ 1 time a week compared to consuming alcohol < 1 time a week.

To measure obesity, height and weight were measured using the scale and transformed BMI (body mass index, kg/m^2^). It was categorized as < 18.5 (underweight), ≥ 18.5 to < 23 (normal), ≥ 23 to < 25 (overweight), ≥ 25 to < 30 (obese I), and ≥ 30 (obese II) based on the Asia–Pacific criteria following the Western Pacific Regional Office (WPRO) 2000^17^.

#### Covariates

The variables of age group, sex, income, and region of residence were categorized following the methods used in our prior study^36^. Age groups were classified as 40–44, 45–49, 50–54…, and 85 + years. The Charlson comorbidity index (CCI) was used for 17 comorbidities as a continuous variable (0 [no comorbidity] through 29 [multiple comorbidities])^37^.

#### Dependent variable

Thyroid cancer was defined using the ICD-10 code (C73). Among the patients, we included only the participants who were treated with any thyroidectomy (Claim code: P4551, P4552, P4553, P4554, or P4561) following the method used in our past study^38^.

### Statistical analyses

Chi-square tests were used to compare the general characteristics between the thyroid cancer group and the control group.

To analyze the odds ratios (ORs) of smoking, alcohol consumption, and obesity in the context of thyroid cancer, conditional logistic regression analysis was used in the matched groups for age, sex, income, and region of residence. In this analysis, crude (simple) and adjusted models (CCI, obesity, smoking status, and frequency of alcohol consumption) were used. In addition, 95% confidence intervals (CIs) were calculated. In these analyses, age, sex, income, and region of residence were stratified. The interaction model between smoking and alcohol consumption is described in Table S3.

For the subgroup analyses, we divided the participants by age and sex (< 55 years and ≥ 55 years, men and women) to confirm these associations according to age and sex. The division of the age groups was determined by the median value of the total number of participants. We used unconditional logistic regression analysis in the subgroup analyses according to smoking status, alcohol consumption, and obesity status. These analyses did not meet the matched structure of the primary design.

Two-tailed analyses were conducted, and P values < 0.05 were considered to indicate significance. The results were statistically analyzed using SPSS version 22.0 (IBM, Armonk, NY, USA) and SAS version 9.4 (SAS Institute Inc., Cary, NC, USA).

## Supplementary information

Supplementary Information.
