# Supplementary material for: Obesity is positively related and tobacco smoking and alcohol consumption are negatively related to an increased risk of thyroid cancer
Source: Sci Rep. 2020 Nov 6;10:19279. doi: 10.1038/s41598-020-76357-y (PMC7648098; doi:10.1038/s41598-020-76357-y)
Supplement: Supplementary file 1 — Supplementary Information. [file 41598_2020_76357_MOESM1_ESM.docx]

**Obesity is positively related and tobacco smoking and alcohol consumption are negatively related to an increased risk of thyroid cancer**

Soo-Youn An, MD^1*^, So Young Kim, MD^2*^, Dong Jun Oh, MD^3^, Chanyang Min, PhD^4,5^, Songyoung Sim, PhD^6^, Hyo Geun Choi, MD^4,7^

^1^Department of Otorhinolaryngology-Head & Neck Surgery, Thyroid/Head & Neck Cancer Center of the Dongnam Institute of Radiological & Medical Sciences (DIRAMS), Busan, Korea

^2^Department of Otorhinolaryngology-Head & Neck Surgery, CHA Bundang Medical Center, CHA University, Seongnam, Korea

^3^Department of Internal medicine, Asan Medical Center, University of Ulsan College of Medicine, Seoul

^4^Hallym Data Science Laboratory, Hallym University College of Medicine, Anyang, Korea

^5^Graduate School of Public Health, Seoul National University, Seoul, Korea

^6^Department of Statistics and Institute of statistics, Hallym University College of Medicine, Chuncheon, Korea

^7^Department of Otorhinolaryngology-Head & Neck Surgery, Hallym University College of Medicine, Anyang, Korea

*These authors are equally contributed in this study

**Table** **S1** The rates of smoking, alcohol consumption, and obesity according to age group and sex.

| Characteristics | | Age group | | | Sex | | |
| --- | --- | --- | --- | --- | --- | --- | --- |
|  | | <55 years old | ≥ 55 years old | P-value | Men | Women | P-value |
| Smoking status | |  |  | <0.001* |  |  | <0.001* |
|  | Nonsmoker or past smoker | 10,605 (91.1) | 12,389 (93.5) |  | 3,627 (70.4) | 19,367 (98.1) |  |
|  | Current smoker | 1,035 (8.9) | 856 (6.5) |  | 1,523 (29.6) | 368 (1.9) |  |
| Alcohol consumption | |  |  | <0.001* |  |  | <0.001* |
|  | < 1 time a week | 9,772 (84.0) | 11,733 (88.6) |  | 3,021 (58.7) | 18,484 (93.7) |  |
|  | ≥ 1 time a week | 1,868 (16.0) | 1,512 (11.4) |  | 2,129 (41.3) | 1,251 (6.3) |  |
| Obesity (BMI, kg/m^2^) | |  |  | <0.001* |  |  | <0.001* |
|  | < 18.5 (underweight) | 206 (1.8) | 205 (1.5) |  | 67 (1.3) | 344 (1.7) |  |
|  | ≥ 18.5 to < 23 (normal) | 4,644 (39.9) | 4,115 (31.1) |  | 1,420 (27.6) | 7,339 (37.2) |  |
|  | ≥ 23 to < 25 (overweight) | 3,234 (27.8) | 3,794 (28.6) |  | 1,587 (30.8) | 5,441 (27.6) |  |
|  | ≥ 25 to < 30 (obese I) | 3,233 (27.8) | 4,606 (34.8) |  | 1,942 (37.7) | 5,897 (29.9) |  |
|  | ≥ 30 (obese II) | 323 (2.8) | 525 (4.0) |  | 134 (2.6) | 714 (3.6) |  |

*Chi-square test. Significance at P < 0.05

**Table** **S2** Adjusted odd ratios (95% confidence interval) of smoking and alcohol consumption in thyroid cancer considering the effect of interaction between smoking and alcohol

| Thyroid cancer | | ORs of thyroid cancer | | | |
| --- | --- | --- | --- | --- | --- |
|  |  | Crude† | P-value | Adjusted†‡ | P-value |
| Smoking status | | 0.60 (0.52-0.69) | <0.001* | 0.47 (0.31-0.73) | <0.001* |
| Alcohol consumption | | 0.79 (0.71-0.88) | <0.001* | 0.67 (0.47-0.94) | 0.020* |
| Smoking*alcohol | | 0.80 (0.75-0.85) | <0.001* | 1.21 (0.91-1.60) | 0.193 |
| Obesity (BMI, kg/m^2^) | |  | <0.001* |  | <0.001* |
|  | < 18.5 (underweight) | 0.74 (0.56-0.98) | 0.037* | 0.75 (0.57-0.99) | 0.041* |
|  | ≥ 18.5 to < 23 (normal) | 1.00 |  | 1.00 |  |
|  | ≥ 23 to < 25 (overweight) | 1.09 (1.00-1.17) | 0.045* | 1.08 (1.00-1.17) | 0.049* |
|  | ≥ 25 to < 30 (obese I) | 1.14 (1.05-1.23) | 0.001* | 1.13 (1.05-1.22) | 0.002* |
|  | ≥ 30 (obese II) | 1.25 (1.05-1.48) | 0.011* | 1.24 (1.04-1.47) | 0.014* |

Conditional logistic regression analysis, Significance at P < 0.05

† Stratified model for age, sex, income, and region of residence.

‡ Adjusted model for Charlson comorbidity index, obesity, smoking state (current smoker compared to non or past smoker), and frequency of alcohol consumption (≥ 1 time a week compared to < 1 time a week), and interaction of smoking state* alcohol drinking habit

Because the interaction smoking state* alcohol consumption did not reach the statistical significance, we did not use this model.

**Table** **S3** Smoking status and alcohol consumption

| Characteristics | | Total participants | | |
| --- | --- | --- | --- | --- |
|  |  | Thyroid cancer (n, %) | Control (n, %) | P-value |
| Smoking status | |  |  | <0.001* |
|  | Nonsmoker | 4,391 (88.2) | 17,141 (86.1) |  |
|  | Past smoker | 313 (6.3) | 1,149 (5.8) |  |
|  | Current smoker | 273 (5.5) | 1,618 (8.1) |  |
| Duration of smoking (total) | |  |  | <0.001* |
|  | Nonsmoker | 4,391 (88.2) | 17,141 (86.1) |  |
|  | < 20 years | 234 (4.7) | 1,006 (5.1) |  |
|  | ≥ 20 years | 352 (7.1) | 1,761 (8.8) |  |
| Current number of cigarettes smoked per day | |  |  | <0.001* |
|  | 0 cigarettes a day | 4,704 (94.5) | 18,290 (91.9) |  |
|  | < 20 cigarettes a day | 178 (3.6) | 1,099 (5.5) |  |
|  | ≥ 20 cigarettes a day | 95 (1.9) | 519 (2.6) |  |
| Alcohol consumption habits | |  |  | <0.001* |
|  | Nondrinker | 3,777 (75.9) | 14,600 (73.3) |  |
|  | < 1 time a week | 611 (12.3) | 2,517 (12.6) |  |
|  | ≥ 1 time a week | 589 (11.8) | 2,791 (14.0) |  |
| Amount of alcohol consumed at one time | |  |  | 0.030* |
|  | < 1 soju bottle | 4,392 (88.2) | 17,340 (87.1) |  |
|  | ≥ 1 soju bottle | 585 (11.8) | 2,568 (12.9) |  |

*Chi-square test. Significant at the level of P < 0.05

Current smoking status was defined as nonsmoker, past smoker, or current smoker. Because the rate of thyroid cancer between nonsmokers and past smokers was not considerably different (P = 0.351 using the Chi-square test, Table S2), we merged nonsmokers and past smokers into one group (nonsmokers or past smokers, Table 1).

The duration of smoking was defined as nonsmokers, < 5 years, 5-9 years, 10-19 years, 20-29 years, and ≥ 30 years. The duration of smoking was recategorized as nonsmokers, < 20 years, and ≥ 20 years.

The current number of cigarettes smoked per day was defined as nonsmokers, < 10 cigarettes, 10-19 cigarettes, 20-39 cigarettes, and ≥ 40 cigarettes and was recategorized as 0 cigarettes per day, < 20 cigarettes per day, and ≥ 20 cigarettes per day.

Alcohol consumption habits were defined as nondrinkers, 2-3 times a month, 1-2 times a week, 3-4 times a week, and ≥ 5 times a week and was recategorized as nondrinkers, < 1 time a week, and ≥ 1 time a week. Because the rate of thyroid cancer between nondrinkers and individuals who consume alcohol < 1 time a week was not substantially different (P = 0.191 using a Chi-square test, Table S2), we merged nondrinkers and individuals who consume alcohol < 1 time a week into one group (< 1 time a week, Table 1).

The amount of alcohol consumed at one time was categorized as < 1 bottle of soju, ~ 1 bottle of soju, > 1 to < 2 bottles of soju, and ≥ 2 bottles of soju. The amount of alcohol consumed was recategorized as < 1 bottle of soju, 1 bottle of soju, and > 1 bottle soju. Soju is the most common alcoholic drink in Korea. Generally, one bottle of soju contains 17.5% alcohol per 360 ml. One bottle of soju is equal to approximately 3.5 bottles of beer.

**Table** **S4** Smoking and alcohol consumption

| Characteristics | | Total participants | | |
| --- | --- | --- | --- | --- |
|  |  | Thyroid cancer (n, %) | Control (n, %) | P-value |
| Smoking status | |  |  | 0.351 |
|  | Nonsmoker | 4,391 (93.3) | 17,141 (93.7) |  |
|  | Past smoker | 313 (6.7) | 1,149 (6.3) |  |
| Alcohol consumption | |  |  | 0.191 |
|  | Nondrinker | 3,777 (86.1) | 14,600 (85.3) |  |
|  | < 1 time a week | 611 (13.9) | 2,517 (14.7) |  |

*Chi-square test. Significance at P < 0.05

**Table** **S5** The distribution of obesity status according to sex

| Characteristics | | Total participants | | |
| --- | --- | --- | --- | --- |
|  |  | Male (n, %) | Female (n, %) | P-value |
| Obesity (BMI, kg/m^2^) | |  |  | <0.001* |
|  | < 18.5 (underweight) | 67 (1.3) | 344 (1.7) |  |
|  | ≥ 18.5 to < 23 (normal) | 1,420 (27.6) | 7,339 (37.2) |  |
|  | ≥ 23 to < 25 (overweight) | 1,587 (30.8) | 5,441 (27.6) |  |
|  | ≥ 25 to < 30 (obese I) | 1,942 (37.7) | 5,897 (29.9) |  |
|  | ≥ 30 (obese II) | 134 (2.6) | 714 (3.6) |  |

BMI: body mass index, kg/m^2^

*Chi-square test. Significance at P < 0.05
